# The impact of Pasifikas in Medicine on Pacific Islander medical student experiences

**DOI:** 10.1371/journal.pone.0340223

**Published:** 2025-12-31

**Authors:** Devon Hori Harvey, Micah Ngatuvai, Siale Vaitohi, Paige E. Faasuamalie, Maegan Tupinio, Lisa H. Smith

**Affiliations:** 1 Department of Ophthalmology, Indiana University School of Medicine, Indianapolis, Indiana, United States of America; 2 Department of Orthopaedic Surgery and Rehabilitation, Texas Tech University Health Sciences Center School of Medicine, Lubbock, Texas, United States of America; 3 Department of Internal Medicine, University of Washington School of Medicine, Seattle, Washington, United States of America; 4 Sidney Kimmel Medical College, Philadelphia, Pennsylvania, United States of America; 5 Department of Anesthesiology, University of Utah School of Medicine, Salt Lake City, Utah, United States of America; 6 Department of Neurology, Indiana University School of Medicine, Indianapolis, Indiana, United States of America; 7 Department of Pediatric Neurology, Riley Children’s Health, Indianapolis, Indiana, United States of America; Instituto Sirio-Libanes de Ensino e Pesquisa, BRAZIL

## Abstract

**Background:**

Pacific Islanders experience significant health disparities. One contributing aspect to these disparities is the lack of racial concordance as Pacific Islanders are underrepresented in the U.S. physician work force. Several factors contribute to this underrepresentation including lack of support systems for Pacific Islander premed and medical students. Pasifikas in Medicine (PiM) is a recently established national student organization founded to provide support for Pacific Islander premed students, medical students, residents, fellows and attending physicians. This study seeks to understand the impact of PiM on medical student experiences.

**Methods:**

An anonymous survey was distributed to the PiM listserv and to Diversity, Equity and Inclusion offices of allopathic and osteopathic medical schools across the U.S. The survey included seven questions for demographic data, ten 5-point ordinal questions to evaluate the impact of PiM on medical student experiences, and three free text questions.

**Results:**

A total of 34 individuals participated in the study with 21 individuals completing the evaluative portion of the survey. Of 28 who responded, 27 (96.4%) were the first in their family to attend medical school, and 25 (89.2%), planned to serve Pacific Islander patient populations in their medical career. For the 10 evaluative questions, 7 scored ≥ 4.0 of of 5.0. Identifying Mentors, Faculty Networking, and Research Opportunities scored less well. Qualitative data was favorable of PiM and demonstrated significant camaraderie, community, and connection to other Pacific Islander physicians and medical students.

**Conclusion:**

Pasifikas in Medicine fills an unmet need by creating a space dedicated to addressing the challenges unique to Pacific Islander students, separate from other minority groups. Improvements to PiM should begin with creating more mentorship opportunities, faculty networking and research opportunities. Additionally, increasing PiM presence nationally and locally within medical schools could further strengthen Pacific Islander medical student experience.

## Introduction

Pacific Islanders, referring to those who originate from the Oceanic regions of Micronesia, Melanesia and Polynesia, experience disparities in health outcomes. Disparities for Pacific Islander populations have been demonstrated in specific disease processes such as chronic kidney disease and breast cancer as well overall health in general [1–3]. Additionally, neonatal mortality rates are significantly higher in Pacific Islander populations when compared to non-Hispanic Whites and other minority groups [4]. Racial and ethnic disparities also have been found to have a staggering economic burden. LaVeist et al. found to be burden attributable to Pacific Islander communities was disproportionately greater than their share of the community [5].

While the root cause of these disparities is multifactorial, one important component is the lack of representation of Pacific Islanders in the physician workforce as racial concordance has been shown to improve patient outcomes [6–8]. In 2022, the AAMC U.S. Physician Workforce Data Dashboard reported that Pacific Islanders comprised only 0.1% of all physicians [9] while Pacific Islanders comprised 0.3% of the U.S. population [10]; thus Pacific Islanders have been recognized as an underrepresented in medicine (URiM) minority group [11].

Medical school admission data from 2024 demonstrated 246 Pacific Islander students who applied to allopathic medical schools in the U.S., comprising 0.5% of the applicant pool [12]. Of the 246 applicants, 94 matriculated in an allopathic medical school, comprising 0.4% of all matriculants [13]. While this result more closely reflects the actual U.S. Pacific Islander population, it is not sufficient to overcome the existing deficit of Pacific Islander physician representation. Additionally, it has been shown that Pacific Islander students exhibit a much higher odds of attrition from medical school, exacerbating this deficit [14].

The challenges to pursue a career in medicine extend beyond the exceptional academic accomplishments one must demonstrate to be considered for admission to medical school. They include exemplifying excellence and uniqueness in aspects such as patient-facing experience, letters of recommendation, shadowing experience, and others. For students who come from URiM backgrounds, these challenges may be compounded by implicit racial bias in medical school admissions [15] and academic disparities that exist in education long before medical school admissions [16]. Therefore, support systems for premed and medical students are particularly vital for URiM students. Established student organizations tailored toward URiM students in the U.S. offer academic and professional guidance along with a sense of belonging that contribute to success and retention [17–19]. Among URiM groups, Pacific Islander students often face unique obstacles due to their small population size in medical schools, which can lead to isolation and limited representation [20–22]. These obstacles are intensified by lack of research specifically addressing Pacific Islander medical students [20].

Pasifikas in Medicine (PiM) is a 501 (c)(3) non-profit organization established in 2019 which is dedicated to support Pacific Islander premed students, medical students, residents, and practicing physicians [23]. At its initiation, PiM consisted of 7 medical students but has since grown and is currently composed of 44 medical trainees, 24 attending physicians and 7 active premed students. Affiliate members include other health care practitioners including physician assistants, nurse practitioners, public health administrators, and psychologist/behavior health specialists. Medical schools from 18 states are represented in PiM, with most representation from UT (8), CA (5), and AZ (4). The most represented medical schools include University of Utah (5), University of California San Diego (4) and University of Arizona (3). Given the limited representation of Pacific Islander students in medical school, students have often undergone their medical training without a Pacific Islander medical community to lean on. PiM is a national organization that seeks to foster a Pacific Islander community in medicine despite widespread geographic separation, increase Pacific Islander representation and increase awareness of the health disparities affecting the Pacific Islander community. In particular, by providing support to Pacific Islander premed and medical students, PiM begins to address the challenges of Pacific Islanders students gaining admission to medical school and the risk attrition after admission. One important aspect of this support is establishing strong mentorship between faculty and medical/premed students. The AAMC Faculty Roster data from 2024 reports that 157 faculty (<0.07%) of the 210,764 total medical school faculty identify as Native Hawaiian/Pacific Islander. [24] While mentorship between Pacific Islander faculty and students is often of great value, mentorship from faculty of all other races/ethnicities will be imperative to support these students. PiM is working to unite Pacific Islander faculty and allies across the country to provide this needed mentorship.

PiM engages with members through different platforms including common channels of communication with other PiM members throughout the U.S., workshops tailored to premed and medical students, regular communications to members with announcements and professional development opportunities, etc. Additionally, Pacific Islander attending physicians and resident physicians often participate in PiM communications and events and provide opportunities for mentorship. Furthermore, PiM hosts a recurring national conference which allows for in-person and virtual correspondence, networking opportunities, community outreach events, as well as opportunities to present research, quality improvement projects and other accomplishments.

This study seeks to investigate current support networks of Pacific Islander medical students and the degree to which participation in PiM supports students. Our research aims to determine whether PiM is successful in providing additional, multifactorial support systems to students during their time in medical school; this will offer new insights into the experiences of this URiM student group.

## Methods

### Study design

This study utilized a cross-sectional design, employing an anonymous online survey (Qualtrics, Provo, UT) created to assess the support and sense of belonging among Pacific Islander medical students. This study was submitted for institutional review board approval and deemed “exempt” by Indiana University (#24092). The recruitment period for this study was from November 1, 2024 to December 31, 2024. Informed consent was obtained in the first question of the survey which was required before accessing the rest of the survey.

The survey consisted of 21 questions: 1 (T/F) informed consent question, 7 anonymous demographic questions, 10 five-point ordinal questions used to evaluate the impact of PiM on Pacific Islander student experience in medical school and 3 free text questions. The five point ordinal scale consisted of the following: 1 - “Disagree,” 2 - “Somewhat Disagree,” 3 - “Neutral,” 4 - “Somewhat Agree,” and 5 - “Agree.” The list of questions is shown in Table 1. No identifiable information was obtained from the participants. Participants were able to withdraw at any time during the survey. Participants were included if they were >18 years of age, current or recently graduated medical students (within 5 years) who self-identify as Pacific Islander. Participants were excluded if they were not a current or previous medical student at a U.S. based medical school or did not finish the survey.

**Table 1:** Pasifikas in Medicine Survey Questions.

Quantitative Questions
1. What stage of medical training are you currently in?
2. Have you heard of the organization “Pasifikas in Medicine?”
3. When did you first learn about the Pasifikas in Medicine?
4. How often did you participate in Pasifikas in Medicine in medical school?
5. Did your medical school have a local organization for Pacific Islander Students?
6. Are you the first to attend Medical School in your family?
7. Do you intend to serve Pacific Islander populations in your career?
8. Do you plan to participate in Pasifikas in Medicine in residency/post-residency?
**Evaluation Questions**
9. Pasifikas in Medicine provided me with opportunities to experience community and connection during medical school.
10. Pasifikas in Medicine provided social support with peers during medical school.
11. Pasifikas in Medicine gave me the opportunity to receive emotional support from peers during medical school.
12. Pasifikas in Medicine helped me identify mentors during medical school.
13. Pasifikas in Medicine provided PEER networking opportunities during medical school.
14. Pasifikas in Medicine provided FACULTY networking opportunities during medical school.
15. Pasifikas in Medicine gave me the opportunity to engage in scholarly activities and/or research during medical school.
16. Pasifikas in Medicine strengthened my ability to improve the health of underserved communities.
17. Pasifikas in Medicine provided enhanced learning opportunities during medical school.
18. Pasifikas in Medicine provided enhanced career opportunities and/or specialty exploration opportunities during medical school.
**Open-ended Questions**
19. Share any personal experiences with Pasifikas in Medicine:
20. What are Pasifikas in Medicine’s strengths?
21. How can Pasifikas in Medicine improve?

List of questions included in anonymous survey which was distributed to Pacific Islander medical students in the United States.

## Pasifikas in medicine survey questions

### Study recruitment

Participants were recruited through two main channels: (1) directly to members of PiM through personal email and to those who follow PiM on social media sites including Instagram and Facebook or (2) emails distributed to Diversity, Equity, and Inclusion (DEI) offices of medical schools across the United States to be communicated to the student body.

### Statistical analysis

Descriptive statistics were generated to summarize participants’ responses based on the 5-point scale associated with the evaluation questions. GraphPad Prism (v. 10.4.2 for macOS, GraphPad Software, Boston, Massachusetts USA) was used to produce statistics and figures.

## Results

A total of 34 participants participated, at least in part, in the survey. Regarding the stage of training, 14 (41.2%) graduated within five years, 8 (23.5%) were in the 4^th^ year, 5 (14.7%) in the 3^rd^ year, 6 (17.6%) in the 2^nd^ year, and 1 (2.9%) in the 1^st^ year. Twenty-eight participants (82.3%) had heard of PiM prior to the survey: 4 (11.8%) during premed, 10 (29%) during 1^st^ year, 8 (23.5%) during 2^nd^ year, 4 (11.8%) during 3^rd^ year, 1 (2.9%) during 4^th^ year, and 1 (2.9%) post-graduation. There were 21 students that participated in PiM with varied levels of participation: 1 (4.7%) student “Almost never participated,” 7 (33.3%) students “Rarely participated,” 8 (38.1%) students “Fairly often participated,” and 5 (23.8%) students “Participated regularly.” There were 16 (47.1%) participants who had a local organization of PiM at their medical school. Of only 28 participants who responded to the following three questions, 27 (96.4%) were the first in their family to attend medical school, 25 (89.2%), planned to serve Pacific Islander patient populations in their medical career, and 23 (82.1%) planned to participate in PiM after medical school graduation.

**Fig 1:**
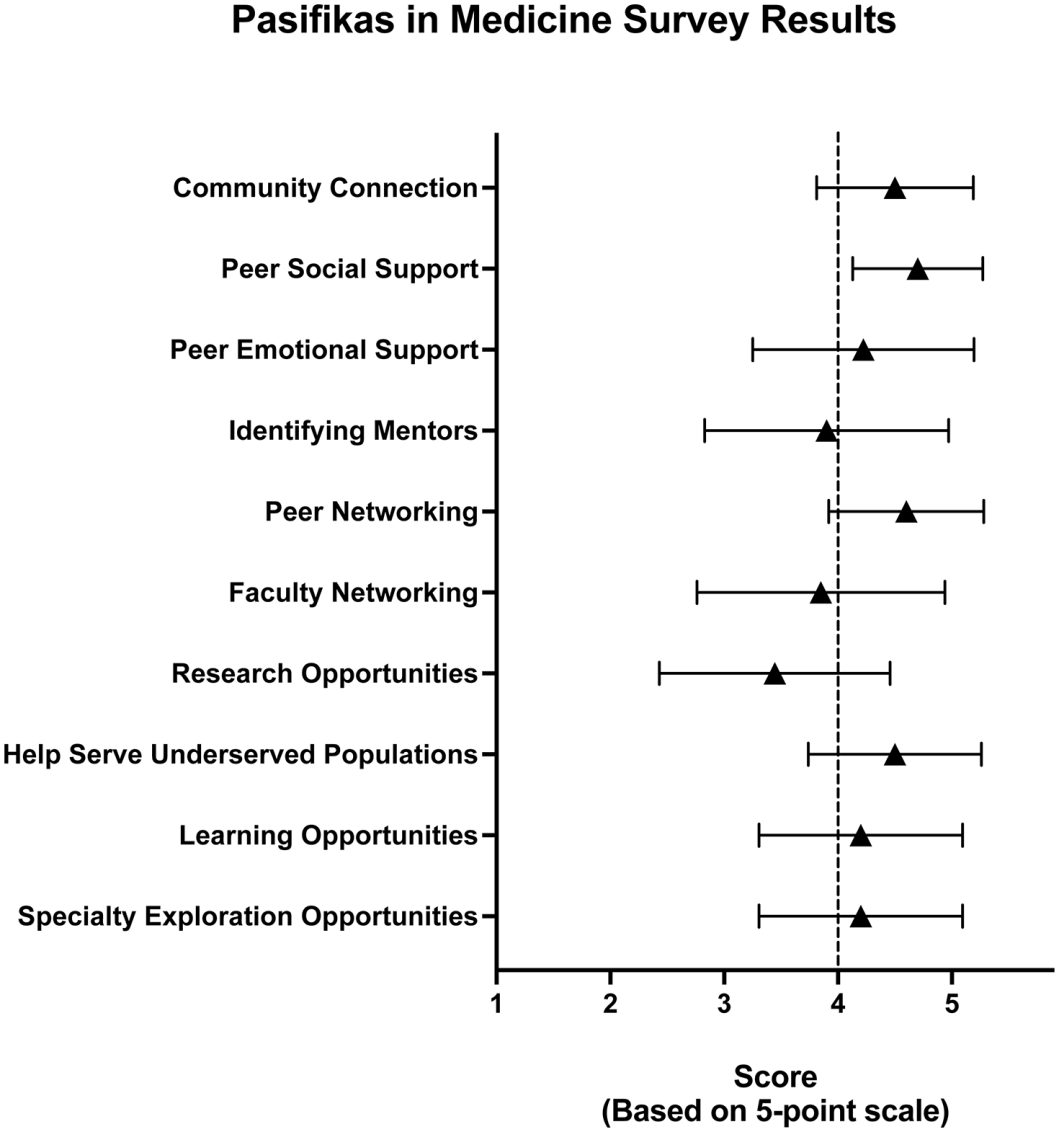
Pasifikas in Medicine Survey Results. Evaluation metric means with standard deviation error bars displayed. All but three (Identifying Mentors, Faculty Networking, and Research Opportunities) metrics scored ≥ 4.0 on the 5-point scale (1 – Disagree, 2 – Somewhat Disagree, 3 – Neutral, 4 – Somewhat Agree, 5 – Agree). N = 21.

Of the total 34 participants, 21 participated in PiM during their medical training and responded to the questions evaluating perceived support and sense of belonging with results represented in Fig 1. In general, results were favorable for all categories except Mentorship, Research, and Faculty Networking.

Participant answers to the free text questions varied in length and content with excerpts shown in Table 2. Reponses to the first question regarding personal experiences with PiM included the following common themes: camaraderie (specifically mentioned in 3 comments), sense of community (specifically mentioned in 3 comments), and establishing meaningful connections with other Pacific Islander students and physicians (specifically mentioned in 3 comments). Participant answers to the second question regarding PiM’s strengths highlighted opportunities to connect with other Pacific Islander medical students given the lack of representation (mentioned in 6 comments). In response to the third question regarding areas of improvement, participants often suggested increasing the size and influence of PiM throughout the country (mentioned in 8 comments).

**Table 2:** Survey Free Text Responses.

Free Text Responses
1) “Share any personal experiences with Pasifikas in Medicine”
“It was great getting to meet people who were so relatable to me in medicine, especially as my medical school did not have many Pacific Islanders.” “Pasifikas in Medicine was an integral part of my medical school life. The organization connected me with friends, helped me find mentors and gave me an opportunity to learn how to lead an organization.” “I think the primary value [of Pasifikas in Medicine] is both the social aspect and identifying mentors with a similar background in a specialty you are interested.”
2) “What are Pasifikas in Medicine’s strengths?”
“Pasifikas in medicine meets an unmet need. Its existence alone and persistent interest/ inclusion of medical students attests to its importance among people coming from Pasifika background.” “Showing representation in a field we’re so rare to be in.”
3) “How can Pasifikas in Medicine improve?”
“Further build the network of [Pasifikas in Medicine].” “Working on more organization within the organization with further involvement of more of our members.” “[Pasifikas in Medicine] is too small. Needs to keep growing.”

Excerpts from participant responses to free text questions are shown above.

## Discussion

The majority of participants were either recent medical schools graduates or were in their fourth year of medical school, though all years (1–4) were represented. The current percentage of Pacific Islander medical school matriculants has been stable at 0.4% since 2016 [13]. Despite there being a similar number of Pacific Islander students each year, we experienced a skewed representation from more senior medical students and graduates. This may suggest that greater efforts should be made to contact and involve students during the first two years of medical school for research on PiM’s influence on medical student experience. Of survey participants, 82% had heard of PiM prior to the survey with the majority having learned of the organization in their first or third year of medical school. Based on recent matriculant data, it is apparent that there are still many Pacific Islander students who did not participate in the survey and have not participated in PiM [13]. This may be due to the fact that PiM is still a relatively new organization with limited presence and medical student awareness across the country. Continued efforts to expand PiM throughout the nation should be made to better serve these students if they choose to participate in the organization.

The large majority of participants reported they were the first in their family to attend medical school and also reported they planned to serve Pacific Islander populations in their career. These results support those of Choi et al. who demonstrated that medical students with close physician relatives were less likely to come from underrepresented backgrounds and were also less likely to report intentions of practicing among the underserved [25]. This is relevant as it has been shown that being from an underrepresented background is a predictor for working with the underserved [25,26]. Supporting Pacific Islander students and other URiM students and organizations dedicated to URiM success, will help to address health disparities of these vulnerable populations.

Our survey results also showed that the majority of participants planned to engage in PiM after graduation from medical school. Just as other student organizations have expanding alumni groups, as PiM continues to grow, the alumni will help create a large network to address the need for more mentorship and support for Pacific Islander students in the future.

Our findings indicate that Pacific Islander medical students consistently found PiM to have positive impacts on their medical school experiences. Based on the 5-point scale, all but three of the 10 outcome measures had a mean score ≥ 4.0. Identifying Mentors, Faculty Networking, and Research Opportunities scored less well and highlight targets for improvement. PiM attempts to engage its members through several different means, which contributes to the favorable scores from participants. National events such as premed workshops, multi-themed guest panels, culturally-sensitive discussions create opportunities for students to connect with one another and find support socially and emotionally in their individual journeys through medicine. Also, connecting through virtual social events and social media allow for informal opportunities to develop relationships with other students from similar backgrounds. Other opportunities for networking exist through service and leadership on any of seven different committees. Additionally, the national conference provides a special opportunity for in-person gathering to connect and network with other students, residents, and faculty from across the nation. Attention to caring for underserved communities is emphasized in PiM through workshops focusing on serving non-English speaking patients, interprofessional collaboration workshops focusing on serving marginalized populations, and events hosted for Pacific Islander communities at large to promote basic healthcare practices such as monitoring blood pressure or discussing diabetes screening. PiM has also grown to include faculty from a variety of medical specialties allowing for exposure and support for career exploration. While efforts are made to connect students with mentors, the limited amount of faculty makes this a challenge. Additionally, PiM provides a research section at the national conference for students to present their work in a formal setting. However, based on the results from this study, further efforts need to be made to provide students with mentors, faculty networking and opportunities to participate in research while in medical school.

The qualitative results were highly supportive of PiM’s impact in medical student experiences and also provided other suggestions to improve PiM as an organization.

These results are consistent with research on other minority medical students, which highlight the value of culturally specific support networks. For instance, Latin medical students benefit from organizations that provide mentorship and a sense of community, improving their overall experience in medical school [17]. The organization Student National Medical Association is made up of majority African American medical students and plays a vital role in helping these students before and after medical school as they enter the physician workforce [18]. Similarly, PiM appears to serve as a significant resource for Pacific Islander students, offering a national network that fosters connection and support. This is particularly critical given the unique challenge of their small population size, which often makes it impractical to form local groups [22]. In many cases, a Pacific Islander student may be the only one of their background at their medical school, intensifying their reliance on national organizations.

The dependence on a national organization like PiM underscores both its strengths and the limitations of local support options. While it effectively bridges gaps for a dispersed population, our findings suggest that additional institutional efforts—such as mentorship programs or local PiM organizations within medical schools—could further enhance support for these students. This dual approach could address both the broad community needs met by PiM and the localized challenges faced at individual schools.

One of the unique challenges faced by Pacific Islander students stems from their relatively small representation in the general population and their even more pronounced scarcity within medicine. This is further complicated by aggregation of Pacific Islander individuals with those of Asian descent. This aggregation has proven detrimental to Pacific Islander individuals, particularly in medical education, as Asian populations—which constitute a distinctly different demographic group—are overrepresented in medicine, thereby skewing data regarding Pacific Islander students and their status as an URiM minority group [20,27]. PiM works to address this need by creating a space specific for Pacific Islander students to confront the unique challenges of this population.

This study has several limitations. First, as Pacific Islanders make up less than 1% of the medical student population, established communication networks were limited. Although we distributed this survey to the national PiM listserv and to DEI components of medical schools via email, it’s possible a large degree of Pacific Islander medical students were unaware of this survey and PiM as an organization. Communication from PiM is primary limited to digital means as the large majority of medical schools do not have a local chapter. Other national groups such as LMSA and SNMA, which have local representatives and chapters, are in many instances able to communicate through other, more personal means such as hosting in-person events or communicating through local chat groups. These are things PiM hopes to incorporate as membership continues to grow across the nation. Furthermore, with a small sample size compared to the total number of Pacific Islander medical student population, the external validity of our results is limited: from 2019 to the time of survey distribution in 2024 there were 455 Pacific Islander students who matriculated into medical school demonstrating that our sample size is but a small portion of the entire Pacific Islander medical student population. However, as PiM experiences continued growth, more robust conclusions will be obtained with more representation from this student group.

The data were collected from a cross-sectional survey and are therefore biased by subjective perceptions and honest feedback from participants. The survey was also anonymous, by which no identifiable information was gathered from the participants and thus, no documentation to confirm that participants did in fact meet the inclusion criteria. One specific limitation encountered in our survey was that several participants did not answer all questions, thus reducing the power of those data. Finally, the majority of participants were engaged in PiM prior to distribution of the survey, which may create biases in the data collected. We attempted to address this with efforts to distribute the survey to all Pacific Islander medical students across the country to also engage those who had not participated but recognize that only a small portion of these students was represented in the survey.

## Conclusion

Our study highlights the critical role of Pasifikas in Medicine (PiM) in supporting Pacific Islander (PI) medical students, a group that seeks greater community and belonging in the face of their small numbers. National organizations like PiM are vital for addressing the needs of such a dispersed population. Improvements to PiM should begin with creating more opportunities to find mentors, to network with faculty members and to obtain research opportunities. Additionally, increasing PiM presence nationally and locally within medical schools could further strengthen Pacific Islander medical student experience. PiM fills an unmet need by creating a space dedicated to addressing the challenges unique to Pacific Islander students, separate from other minority groups. Finally, PiM has the potential to be a valuable resource to increase the representation of Pacific Islander individuals in medicine and among all different medical specialties.

## Supporting information

S1 Dataset Dataset of PiM survey results. (XLSX)
